# A Five-Year (2016-2020) Trend Analysis of Malaria Surveillance Data in Oromia Regional State, Ethiopia

**DOI:** 10.1155/2023/5278839

**Published:** 2023-08-05

**Authors:** Zalalem Olani, Samrawit Solomon, Zalalem Kaba, Haile Bikila

**Affiliations:** ^1^Department of Public Health, Saint Paul's Millennium Medical College, Addis Ababa, Ethiopia; ^2^Department of Public Health, Institute of Health Sciences, Saint Paul's Millennium Medical College, Addis Ababa, Ethiopia; ^3^Oromiyaa Regional Health Bureau, Addis Ababa, Ethiopia; ^4^Department of Public Health, Institute of Health Sciences, Wollega University, Nekemte, Ethiopia

## Abstract

**Background:**

Continuous malaria surveillance data analysis plays a significant role in monitoring trends over time and evaluating the effectiveness of malaria prevention and control programs. Hence, this study was part of an effort to achieve this goal. This study's main aim was to analyse five years (2016-2020) of malaria surveillance data in the Oromia Region, Ethiopia.

**Methods:**

A descriptive cross-sectional study design was used to analyse the five-year (2016-2020) trend of malaria cases in the Oromia Regional State, Ethiopia.

**Results:**

A total of 5,843,373malaria suspected cases were reported during the five-year period. Among the total reported cases, 727,738 were a total of both clinical and parasitological confirmed cases. The average total malaria annual parasite incidence (API) was 4 per 1,000 persons. The highest malaria cases were observed during the spring and summer seasons. *Conclusions and Recommendation*. Trends of total clinical and confirmed malaria cases decreased from year to year except for the recent year with an exceptional variability in 2019. The highest or peak of malaria cases was observed during spring season (September-November). Malaria indicator-based performance plans and achievements should be regularly and strictly reviewed and evaluated at each level.

## 1. Background

Malaria is a life-threatening protozoan disease caused by plasmodium parasites that are transmitted to people through the bites of infected female Anopheles mosquitoes. It is commonly caused by four species of the genus *Plasmodium* including *Plasmodium vivax* (*PV*), *Plasmodium falciparum* (*PF*), *Plasmodium ovale* (*PO*), *Plasmodium malariae* (*PM*), and *Plasmodium knowlesi* (*PK*) that are known to infect human beings. Out of the plasmodium species which causes malaria, *PF* is the deadliest parasite in terms of its morbidity and mortality and the most prevalent malaria parasite in sub-Saharan Africa accounting 99% for malaria cases in 2016, whereas *PV* spread geographically in the most densely populated regions [1]. Globally, 84 malaria-endemic nations (including French Guiana) reported an expected 247 million cases of malaria in 2021, up from 245 million in 2020, with the majority of this rise occurring in nations in the WHO African Region. Between 2019 and 2021, an estimated additional 13.4 million cases were attributed to disruptions during the COVID-19 pandemic [2]. The World Health Organization (WHO) African estimated over 200 million cases of malaria each year, with 90% of cases and 90% of deaths to occur in Africa [3].

In Ethiopia, 68% of the areas are endemic for malaria, and 60% of the country population are prone for infection of malaria. Mortality and morbidity attributed to malaria that declined significantly from 2015/16 to 2018/19, though morbidity has increased in 2019/2020. Death due to malaria has declined by 67% from 0.9/100,000 population to 0.3/100,000 population at risk between 2016 and 2019. Similarly, the annual parasite incidence (API) has declined by 37% from 19/1,000 population to 12/1,000 population in the same year. The number of confirmed malaria cases has reduced by 47% between 2016 and 2019. The trend of the number of malaria cases was diagnosed over the last five years; in Ethiopia, health sector transformation plan phase one (HSTP-I period) showed that malaria was consistently decreasing from 2016 until 2019, but it has increased in 2020. In 2020 (2012 Ethiopian fiscal year), 1,509,182 total malaria cases (clinical and laboratory confirmed) were diagnosed, among which 1,398,750 (93%) were laboratory-confirmed malaria cases. The number of total malaria cases in 2012 shows an increment by 515,183 cases (increased by 52%) from the 2011 EFY (Ethiopian fiscal year) malaria cases. According to 2012 Ethiopian fiscal year (EFY) federal ministry of health (FMOH) annual report in Oromia Regional State, around 53% of total populations are at risk of malaria case, 8% incidence rate, and 0.37 deaths out of 100,000 populations [4].

To realistically embark on the road towards malaria elimination, timely provision of accurate malaria surveillance data is necessary. In the context of the massive scale up of malaria interventions, there is increasing recognition that the current capacity of routine malaria surveillance, conducted in most African countries through integrated health management information systems (HMIS), is inadequate; indicators are poorly defined; data reporting and completeness are often of unknown quality; and timeliness can be extremely variable. Further, there is limited capacity for data analysis, data interpretation, and action [5].

**Figure 1:**
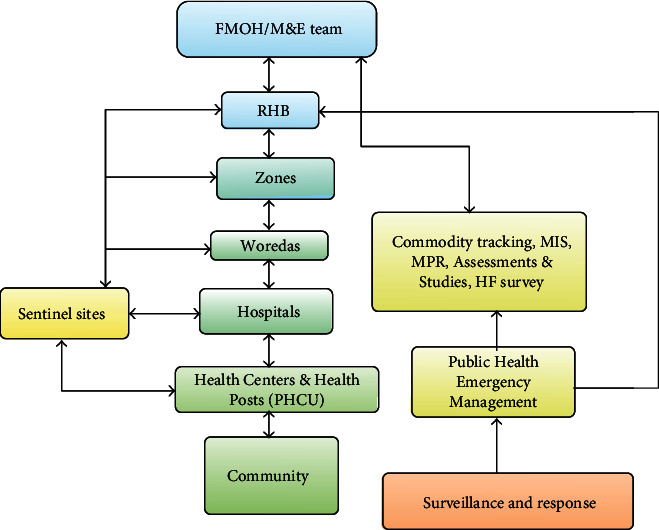
Overview of data source, flow, and use in malaria M&E system (adapted from the National Malaria Program Monitoring and Evaluation Plan 2014-2020).

To create a malaria-free nation, the government has set a big goal of eliminating malaria by 2030 [3]. To achieve the set goal, all concerned bodies in the country have shown a strong commitment. Hence, the country has been able to achieve about 50% overall malaria reduction goal by 2015. This remarkable achievement was attained mainly due to the implementation of intensive interventional strategies such as indoor residual spraying, use of bed nets, and combination chemotherapy. However, in some endemic areas of the same country, this reduction has not yet been achieved, and it is a major cause of illness and death [5]. In the past few years, the country has achieved substantial reduction of the burden of malaria from populations at risk. Despite the above promising outcomes, Ethiopia still needs to do much for malaria elimination. And the aim of this surveillance data analysis is to provide factual information, on the trend of malaria case, and locate malaria cases by place, person, and time to allocate resource and act timely for elimination. Besides, the information generated from the analysis of surveillance data is important to initiate public health action, which helps to identify the available gaps in the surveillance system (Figure 1).

## 2. Methods and Materials

### 2.1. Study Area and Period

It was conducted in Oromia Regional State, Ethiopia. It is one of the ten regions in Ethiopia. Addis Ababa is the capital city of the region. Currently, it consists of 21 administrative zones and 19 towns. The region is bordered by the Somali Region to the east; the Amhara Region, the Afar Region, and the Benishangul-Gumuz Region to the north; Dire Dawa to the northeast; the South Sudanese state of Upper Nile, Gambella Region, Southern Nations, Nationalities, and Peoples' Region, and Sidama Region to the west; the Eastern Province of Kenya to the south; and the Addis Ababa as an enclave surrounded by Oromia Special Zone Surrounding Finfinne in its center and the Harari Region as an enclave surrounded by East Hararghe in its east. Malaria surveillance data or Oromia Region reported on a weekly or monthly or yearly basis for the last five years (2016-2020) was analysed and interpreted from March 22 to May 15, 2021.

### 2.2. Study Design

A descriptive cross-sectional study design was employed to assess a five-year (2016-2020) trend analysis of malaria surveillance data in Oromia Regional State, Ethiopia.

### 2.3. Population

#### 2.3.1. Source Population

The source population was any people living in Oromia Region that have sought health care services within the Oromia Regional State from January 2016 to December 2020.

#### 2.3.2. Study Population

All reported cases of malaria in Oromia Region health facilities between January 2016 and December 2020 who satisfy the inclusion criteria are the study population.

### 2.4. Inclusion and Exclusion Criteria

A total of five-year period confirmed and clinically treated cases for malaria were included in the study, and all incomplete data were excluded.

### 2.5. Study Variables

- Months
- Zone
- Confirmed and clinical malaria case
- Suspected fever cases examined for malaria case
- Confirmed case PF malaria cases
- Confirmed case PV malaria cases
- Inpatient case due to malaria
- Inpatient death due to malaria
- Clinical malaria case

### 2.6. Data Collection Tools and Procedures

Five-year secondary data were obtained from Ethiopian Public Health Institute (EPHI) PHEM weekly surveillance data of 2016-2019. Variables such as zones, woreda, clinical and confirmed, inpatient, outpatient, PF and PV, malaria suspected cases, and malaria deaths were included in the database.

### 2.7. Data Processing and Analysis

All data were checked for completeness and cleaned for any inconsistencies to analyse. The data were entered and analysed with Microsoft Office Excel 2010. Descriptive statistics were used to show the trends of malaria transmission in terms of seasons, years, and species of malaria parasite. The analysed data were presented using tables and figures.

### 2.8. Data Quality Control

The collected data were checked for completeness, accuracy, clarity, and consistency throughout the data collection period to maintain the quality of data.

### 2.9. Ethical Considerations

Ethical clearance was secured by writing a formal letter from St. Paul's Hospital Millennium Medical College (SPHMMC) to Ethiopian Public Health Institute (EPHI) and then submitted to Public Health Emergency Management (PHEM) surveillance team. Official permission was obtained from concerned authorities of the health office. The surveillance officers and other health professionals were informed about the objective and purpose of the surveillance data analysis to cooperate with the data analyser throughout data collection and analysis. All methods were performed according to the national and international public health research guidelines and regulations.

### 2.10. Operational Definitions

- Suspected malaria case: clinical diagnosis of malaria is made in a patient who has fever or history of fever in the last 48 hours and lives in malaria-endemic areas or has a history of travel within the last 30 days to malaria-endemic areas
- Confirmed malaria case: a suspected case of malaria in which malaria parasites have been verified by microscopy or RDT (rapid diagnostic test)
- Annual parasite incidence: total number of positive slides for malaria parasite in a year ^∗^1,000/population at risk
- Malaria outbreaks: crossing the norm line or doubling the number of malaria cases compared to the prior year of reported WHO epidemic week
- Slide positivity rate/SPR: the number of laboratory-confirmed malaria case per 100 cases examined cases

## 3. Results

**Figure 2:**
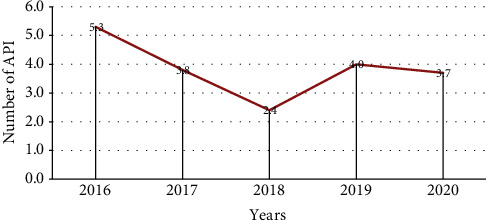
Trends of API from January 2016 to December 2020, Oromia Regional State, Ethiopia.

The Oromia Regional State five-year (2016-2020) malaria surveillance data were systematically analysed and verified. It was indicated that the total number of 5,843,373 suspected malaria cases was reported in the region from January 2016 to December 2020. Among the reported cases, 727,738 (clinically confirmed cases: 15,655, 2.2%, and parasitological confirmed cases: 712,083, 97.8%). Malaria detection rate from total suspected fever examined by RDT or microscopy and clinical case was 12% (712,083/5,843,373). During the study period (2016-2020), API was decreased by 5.3 per 1,000, and 3.8 per 1,000 and 2.4 per 1,000 during 2016 and 2018, respectively. Conversely, it showed an increment during 2019 by 4 and slight decrement by 3.7 in the year 2020. Among the parasitological confirmed cases, PF constitutes 68.2% (485,350/712,083) while the rest 31.8 (226,733/712,083) were covered by PV (Table 1 and Figure 2).

### 3.1. Description of Malaria Morbidity and Mortality by Time

**Figure 3:**
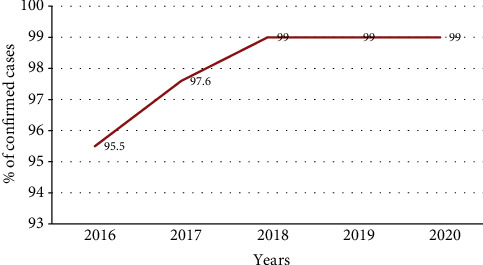
Trends of confirmed malaria cases in percentage from 2016 to 2020, Oromia Regional State, Ethiopia.

From the total cases of malaria reported during study period, those cases confirmed by microscopy or RDT were increasing from 95.5% in 2016 to 97.6% in 2017 and then slightly constant from 2019 to 2020 by 99% (Figure 3).

### 3.2. Trends of Malaria Case by Species

**Figure 4:**
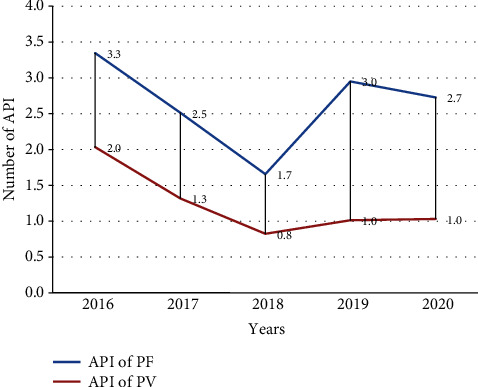
Trends of positive malaria cases with species from 2016 to 2020, Oromia, Ethiopia.

Regarding the identified plasmodium species, both species of plasmodium were reported in each year, with PF being the predominant species in the study area. From the total of 712,083 outpatient confirmed cases reported during the five years (2016-2020), 68.2% (485,350) of the case were due to PF, and the rest 22,673 (31.8%) were due to PV. PF and PV were the only species in the study area, where PF accounted for 68.2% of the overall incidence, followed by PV constituting 31.8%. The incidence of PF cases was decreasing beginning from 2016 to 2018 by 3.3 per 1,000, 2.5 per 1,000, and 1.7 per 1,000 persons and by the year 2019 shows a slight increment by 3 per 1,000, and by the year 2020, it shows a slight decrement 2.7 per 1,000 persons, respectively. The incidence of PV was 2 per 1,000, 1.3 per 1,000, and 0.8 per 1,000 persons in 2016-2018, and from 2019 to 2020, the case showed relatively constant by 1 per 1,000 persons. In the past five-year trend, PF was more dominant over PV (Figure 4).

**Figure 5:**
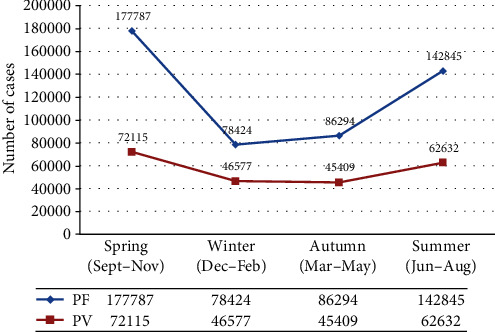
The distribution of *PF* and *PV* species in different seasons from 2016 to 2020, Oromia Regional State, Ethiopia.

Even though malaria case occurred in all seasons, the incidence had fluctuating trend across four seasons over the last five years. The highest malaria cases were observed during two seasons: the first one was in spring by 249,902 (35%) and the second one was in summer by 205,477 (28.8%). On the other hand, the lowest cases were observed during winter by 125,001 (17.5%). As far as the species are considered, higher number of cases of PF and PV was observed during spring (177,787 (36.6%) and 72,115 (30.1%)) and summer (142,845 (29.7%) and 62,632 (27.6%)), respectively. However, the minimum numbers of PF (78,424 (16%)) in spring and slightly decrement of PV cases by 45,409 (20.1%) were observed during autumn (Figure 5).

**Figure 6:**
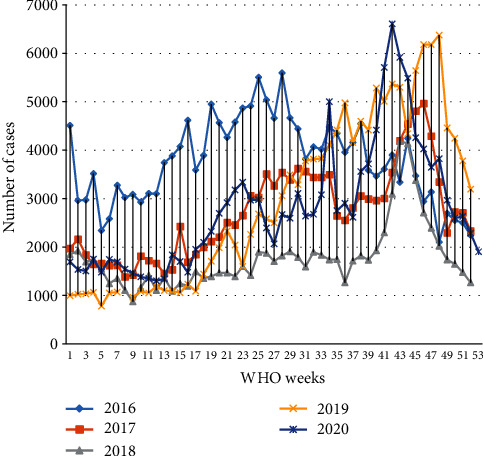
Trends of malaria in WHO epidemic weeks from January 2016 to December 2020, Oromia Regional State, Ethiopia.

The peak weekly incidence rate in the past five years was observed on week 41 of 2020 with 166 cases per 100,000 population, and the lowest weekly incidence rate was reached on week 5 of 2018 with 19 cases per 100,000 population (Figure 6).

**Figure 7:**
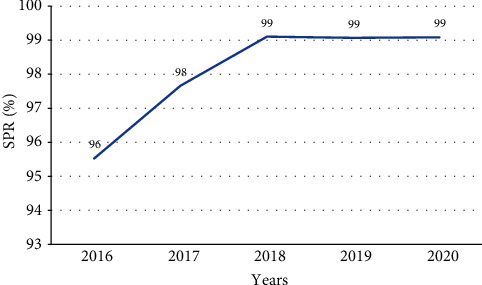
Trends of malaria SPR from January 2016 to December 2020, Oromia Regional State, Ethiopia.

From a total of 727,738 suspected fever cases examined by RDT or microscopy, 712,083 patients were positive for plasmodium species from January 2016 to December 2020, and then, there was an average malaria slide positivity rate (SPR) of 96%. Malaria SPR was increased from 2016 to 2018 by 96%, 98%, and 99%, respectively, and kept constant in 2019 and 2020 by 99% (Figure 7).

### 3.3. Malaria Morbidity and Mortality by Place

*PF* was the most commonly reported species accounted for 485,350 (68%) of the cases while *PV* was reported among 226,733 (32%) of the positive results. Among all zones and towns of Oromia Region, *PF* is the highest in magnitude in 19 zones and towns and *PV* is highest in 18 zones and towns of Oromia Region. The magnitude of the species was different among zones and towns of the region. Malaria cases were less common in 8 towns among 18 of them included in the study (Table 2).

**Figure 8:**
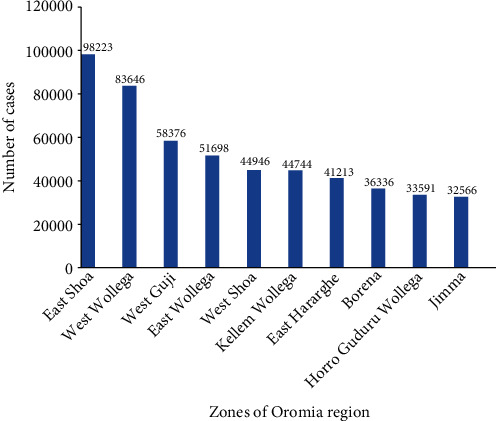
Top ten zones of Oromia Regional State by total confirmed malaria cases from January 2016 to December 2020, Ethiopia.

East Shoa recorded the highest total confirmed malaria case with 98,223; West Wollega, West Guji, East Wollega, West Shoa, Kellem Wollega, East Hararghe, Borena, Horro Guduru Wollega, and Jimma are recorded to have the respective rank next to East Shoa (Figure 8).

More malaria deaths were observed by the analysis of past five years in Oromia Regional State. West Arsi, West Wollega, West Hararghe, and Kellem Wollega Zones had more number of deaths, respectively, than the other zones (Table 3).

**Figure 9:**
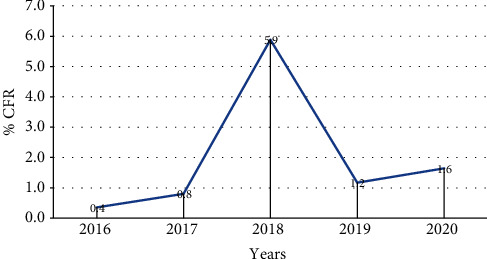
Trends of malaria CFR in Oromia Regional State from 2016 to 2020, Ethiopia.

A total of 5,180 malaria inpatient cases and 63 deaths were reported for the last five years from January 2016 to December 2020. The case fatality rate (CFR) was high in 2018 with 5.9%. CFR showed consistent increment from 2016 to 2018, by 0.4%, 0.8%, and 5.9%, respectively. It was 1.2% and 1.6% during 2019 and 2020, respectively (Figure 9).

## 4. Discussion

The present study attempted to assess the trend of malaria incidence in Oromia Regional State from January 2016 to December 2020, Ethiopia. According to the last five years of malaria surveillance data of Oromia Regional State, a total of malaria cases by Zone indicated that high number of cases was found in East Shoa, West Wollega, West Guji, East Wollega, Kellem Wollega, East Hararghe, Borena, Horro Guduru Wollega, and Jimma. Besides, more malaria deaths were observed in West Arsi, West Wollega, West Hararghe, Kellem Wollega, Horro Guduru Wollega, Ilu Aba Bora, West Guji, Finfinne Zuria, East Wollega, and West Shoa, respectively. Malaria cases were less common in this study. This may be due to geographical locations of the towns, limited number of breeding sites, having adequate knowledge of malaria, improved housing conditions, and socioeconomic conditions.

Trends of total clinical and confirmed malaria cases were decreased from year to year except for the recent one year with an exceptional variability in 2019. The highest or peak of malaria cases was observed during spring (September-November). In this assessment, a total of 727,738 confirmed and clinical malaria cases were reported within the last five-year period from January 2016 to December 2020 with a mean annual occurrence of 145,548 cases. It could be an important indicator for existence of malaria burden in the study area, which seems to need due attention towards malaria intervention during this critical period of the national striving towards malaria elimination in the year 2030 [6].

The general declined number of clinical malaria cases was seen in the study (2.15%). The same study conducted in Azebo in northern Ethiopia from 2011 to 2016 indicated that the number of clinical malaria cases declined [7]. Another study done in Oromia Regional State in Batu town from 2012 to 2017 indicated that the number of clinical cases declined [8]. This may be due to the improved provision of diagnostic facilities like RDT and diagnostic microscope for health facilities.

*PF* and *PV* were the dominant cause of malaria in Oromia Regional State from 2016 to 2020, accounting for 68.2% and 31.8%, respectively. *PF* is the most prevalent malaria parasite in the WHO African Region, accounting for 99.7% of the estimated malaria cases in 2018, as well as in the WHO Southeast Asian Region (50%), the WHO Eastern Mediterranean Region (71%), and the WHO Western Pacific Region (65%) [5]. This assessment was in line with the study conducted in Ethiopia during 2016, which revealed that *PF* is the leading cause of malaria in Ethiopia by 60% while *PV* causes 40% of malaria in Ethiopia [9]. A five-year trend analysis of malaria prevalence in Dembecha Health Center, West Gojjam zone, northwest Ethiopia 2016, indicated the same condition for the causative agents of malaria which is *PF* causing about 68.2% and *PV* causing 26.3% [8].

Unlike our study result showing a difference on proportions of the species compared to other studies, “Malaria epidemiology and interventions in Ethiopia from 2001 to 2016” shows that *PF* and *PV* coexist, accounting for 60 and 40% of all malaria cases, respectively [10]. In another study done in “Oromia Regional State, in Adama City,” out of the 6862 malaria cases reported from OPD data from 2013/14 to 2017/18 in retrospective study, 61% was *PV* and 39% was *PF* [11].

In this assessment, malaria cases were reported in all the four seasons of Ethiopia. The peak of malaria incidence occurs during spring (September, October, and November), and the second peak of malaria incidence was observed during summer (June, July, and August). This finding was in line with the studies conducted in Bale zone, North West Tigray, East Wollega zone, and Wolkite health center [6, 8, 12, 13].

API was decreased by 5.3 per 1,000, 3.8 per 1,000, and 2.4 per 1,000 during 2016 and 2018, respectively. Conversely, it showed an increment during 2019 by 4 and slight decrement by 3.7 in the year 2020 per 1,000 population, respectively. In 2020, the FMOH updated the country's malaria risk strata based upon malaria API; the area with malaria transmission risk by ≤5 cases/1,000 population/year is classified as very low API, so Oromia Regional State is categorized under low-risk classification according to FMOH [3]. Proportion of parasitological confirmation treatment rate sharply increases from 95.5% to 99% during the last five years (2016-2020), respectively. Unlikely, the clinical malaria treatment rate decreases in the same study year from 4.5% to 0.99%. This retrospective study was unlike with a five-year trend analysis of malaria prevalence in Mankush Health Center, Guba district, Benishangul-Gumuz Regional State, and western Ethiopia from 2014 to 2018, which shows dramatic decrement of parasitological confirmation treatment rate from 85% to 51% [14]. This controversial issue might be due to improper supply of diagnostic materials and lack of well-trained health professionals on the diagnostic materials.

A total of 5,180 malaria inpatient cases and 63 deaths were reported for the last five years (January 2016-December 2020). The CFR in the same period was fluctuating. It was high in 2018 with 5.9%. CFR showed consistent increment from 2016 to 2018, by 0.4%, 0.8%, and 5.9%, respectively. It was 1.2% and 1.6% during 2019 and 2020, respectively. This was in agreement with the study done in Bale zone from 2010 to 2017 which also supports our findings describing that the annual number of malaria death is fluctuating by 0%, 1%, and 2% in 2010, 2011, and 2012, respectively, and 0% from 2013 to 2016 and 6% in 2017 [15].

### 4.1. Limitations of the Study

The data we analysed lacks important personal variables such as age, sex, and pregnancy status of females; due to this fact, it is difficult to analyse the impact of malaria by age and sex.

## 5. Conclusion and Recommendation

### 5.1. Conclusion

Trends of total clinical and confirmed malaria cases were decreased from year to year except for the recent one year, with an exceptional variability in 2019. The highest peak of malaria cases was observed during spring (September-November). The highest cases were seen in East Shoa, West Wollega, West Guji, East Wollega, Kellem Wollega, East Hararghe, Borena, Horro Guduru Wollega, and Jimma. Malaria-related death happened within the study period in the following zones of Oromia Regional State: West Arsi, West Wollega, West Hararghe, Kellem Wollega, Horro Guduru Wollega, Ilu Aba Bora, West Guji, Finfinne Zuria, East Wollega, and West Shoa. Malaria is an important public health problem in the study area, with a predominance of PF and PV infection. It could be an indicator for existence of malaria burden in the study area, which seems to need due attention towards malaria intervention during this critical period of the national striving towards malaria elimination in the year 2030.

### 5.2. Recommendation

Depending on our assessment results, we would like to forward the following recommendations to EPHI, Oromia Regional Health Bureau (ORHB), SPMMC, and other concerned bodies or stakeholders:

(i) Strong emphasis should be given to the analysis of malaria surveillance and use for decision-making at country level as well as regional level, with an effort to avert higher cases of malaria in the study area; performance plans and achievements should be regularly and strictly reviewed and evaluated at each level
(ii) Zones with the highest cases like East Shoa, West Wollega, West Guji, East Wollega, Kellem Wollega, East Hararghe, Borena, Horro Guduru Wollega, and Jimma should be given special attention
(iii) Zones with malaria-related deaths like West Arsi, West Wollega, West Hararghe, Kellem Wollega, Horro Guduru Wollega, Ilu Aba Bora, West Guji, Finfinne Zuria, East Wollega, and West Shoa require special intervention
  - We recommend the zonal and woreda health offices, health workers, health extension workers, and the community to strength early detection and treatment of malaria cases at all level to decrease malaria inpatient and CFR which is part of strengthening surveillance data analysis
  - The regional health bureau and the district health office should plan to do further study to know the cause of high incidence of *PF* malaria species, malaria inpatient, and malaria CFR for prevention and control of malaria to come to elimination
  - It is better if personal variables such as age, sex, and pregnancy are incorporated in PHEM reporting format for detailed analysis of the impact of malaria on personal characteristics and to identify the risk groups
  - The PHEM officers at district and health facility level should strengthen documentation and handling of malaria data by species in all kebele of the district and analyse the data and alert the community before occurrence of outbreak during pick transition seasons

## Figures and Tables

**Table 1:** Last five years summarized malaria reported data from January 2016 to December 2020 of Oromia Regional State, Ethiopia.

Years	Total population (population at risk)	Total malaria suspected fever examined	Total malaria parasitological confirmed and clinical (that is, both *PF* and *PV*)	Total positive malaria by RDT or microscopy outpatient cases	
				*PF*	*PV*
2016	34,895,497	1,252,144	196,451	116,740	70,919
2017	35,856,743	1,152,288	140,526	90,045	47,196
2018	36,839,051	1,099,258	92,129	60,979	30,325
2019	37,831,920	1,244,753	151,319	111,607	38,308
2020	38,865,436	1,094,930	147,313	105,979	39,985
Grand total		5,843,373	727,738	485,350	226,733

**Table 2:** Malaria cases distribution by species by from January 2016 to December 2020 in zones and towns of Oromia Regional State, Ethiopia.

S. no	Lists of zones and towns in the region	Total parasitological confirmed case	Total five years of PF		Total five years of PV	
			Frequency (*N*)	Percentage (%)	Frequency (*N*)	Percentage (%)
1	Adama special town	5,545	2,167	39	3,378	61
2	Ambo town	154	60	39	94	61
3	Arsi	20,642	13,070	63	7,572	37
4	Asella town	425	86	20	339	80
5	Bale	2,086	1,794	86	292	14
6	Bishoftu town	2,916	822	28	2,094	72
7	Borena	36,336	28,813	79	7,523	21
8	Buno Bedele	14,971	11,998	80	2,973	20
9	Burayu town	279	70	25	209	75
10	Dukem town	930	384	41	546	59
11	East Hararghe	41,213	36,214	88	4,999	12
12	East Shoa	98,223	59,751	61	38,472	39
13	East Wollega	51,698	39,597	77	12,101	23
14	Finfine Zuria	3,545	1,872	53	1,673	47
15	Gelan town	83	18	22	65	78
16	Guji	29,240	21,196	72	8,044	28
17	Holeta town	106	42	40	64	60
18	Horro Guduru Wollega	33,591	21,386	64	12,205	36
19	Ilu Aba Bora	19,521	12,842	66	6,679	34
20	Jimma	32,566	21,364	66	11,202	34
21	Jimma special town	4,785	1,189	25	3,596	75
22	Legetafo Legedadi town	156	54	35	102	65
23	Modjo town	1,514	663	44	851	56
24	Nekemte town	2,234	989	44	1,245	56
25	North Shoa	11,511	7,841	68	3,670	32
26	Kellem Wollega	44,744	30,125	67	14,619	33
27	Robe town	1	1	100	0	0
28	Sebeta town	1,605	372	23	1,233	77
29	Shashamane town	10,480	3,308	32	7,172	68
30	Southwest Shoa	22,799	10,608	47	12,191	53
31	Sululta town	66	16	24	50	76
32	West Arsi	19,023	10,044	53	8,979	47
33	West Guji	58,376	40,653	70	17,723	30
34	West Hararghe	9,110	8,032	88	1,078	12
35	West Shoa	44,946	26,796	60	18,150	40
36	West Wollega	83,646	70,668	84	12,978	16
37	Waliso town	3,017	445	15	2,572	85
Grand total		712,083	485,350	68	226,733	32

**Table 3:** Total malaria deaths by zones and towns of Oromia Regional State from January 2016 to December 2020, Ethiopia.

Zone and towns	Total number of malaria death within the last five years (2016-2020)
West Arsi	20
West Wollega	13
West Hararghe	9
Kellem Wollega	7
Horro Guduru Wollega	4
Ilu Aba Bora	4
West Guji	3
Finfine Zuria	1
East Wollega	1
West Shoa	1
Other zones or towns in Oromia Region	0
Grand total	63

## Data Availability

The finding of this study was generated from the data collected and analysed based on the stated methods and materials. The original data supporting this finding are available from the corresponding author on reasonable request.

## Acronyms

API: Annual parasite incidence
CFR: Case fatality rate
CSA: Central Statistical Agency
EPHI: Ethiopian Public Health Institute
FMOH: Federal Ministry of Health
ORHB: Oromia Regional Health Bureau
PF: Plasmodium falciparum
PHEM: Public Health Emergency Management
PV: Plasmodium vivax
RDT: Rapid diagnostic test
SPMMC: Saint Paul's Millennium Medical College
SPR: Slide positivity rate
WHO: World Health Organization.
